# No Difference in the Incidence of Malaria in Human-Landing Mosquito Catch Collectors and Non-Collectors in a Senegalese Village with Endemic Malaria

**DOI:** 10.1371/journal.pone.0126187

**Published:** 2015-05-12

**Authors:** Amélé N. Wotodjo, Jean-François Trape, Vincent Richard, Souleymane Doucouré, Nafissatou Diagne, Adama Tall, Ousmane Ndiath, Ngor Faye, Jean Gaudart, Christophe Rogier, Cheikh Sokhna

**Affiliations:** 1 Unité de Recherche sur les Maladies Infectieuses et Tropicales Émergentes, IRD (Institut de Recherche pour le Développement), UMR (Unité Mixte de Recherche) 198, UM63, CNRS (Centre national de la recherche scientifique) 7278, INSERM (Institut national de la santé et de la recherche médicale) 1095, Aix-Marseille Université, Campus UCAD (Université Cheikh Anta Diop de Dakar)-IRD, BP 1386, CP 18524, Dakar, Sénégal; 2 Institut de Recherche pour le Développement, Laboratoire de Paludologie, Dakar, Sénégal; 3 Institut Pasteur de Dakar, Unité d’Épidémiologie, Dakar, Sénégal; 4 Institut Pasteur International Network, G4 Leader Group, Institut pasteur of Bangui, Bangui, Central African Republic; 5 Université Cheikh Anta Diop de Dakar, Faculté des Sciences et Techniques, Laboratoire de Parasitologie, Dakar, Sénégal; 6 Aix-Marseille Université, UMR (Unité Mixte de Recherche) 912, SESSTIM (Sciences Économiques & Sociales de la Santé & Traitement de l’Information Médicale), (INSERM-IRD-AMU), Marseille, 13005, France; 7 Institut Pasteur de Madagascar, Antananarivo, Madagascar; 8 Institut de Recherche Biomédicale des Armées, Brétigny sur Orge, France; 9 Aix Marseille Université, Unité de Recherche sur les Maladies Infectieuses et Tropicales Émergentes, UM 63, CNRS (Centre national de la recherche scientifique) 7278, IRD 198, INSERM 1095, Marseille, France; Oswaldo Cruz Foundation, BRAZIL

## Abstract

**Background:**

The human landing catches is the gold standard method used to study the vectors of malaria and to estimate their aggressiveness. However, this method has raised safety concerns due to a possible increased risk of malaria or other mosquito-borne diseases among the mosquito collectors. The aim of this study was to evaluate the incidence of malaria attacks among mosquito collectors and to compare these results with those of non-collectors in a Senegalese village.

**Methods:**

From July 1990 to December 2011, a longitudinal malaria study involving mosquito collectors and non-collectors was performed in Dielmo village, Senegal. During the study period, 4 drugs were successively used to treat clinical malaria, and long-lasting insecticide-treated nets were offered to all villagers in July 2008. No malaria chemoprophylaxis was given to mosquito collectors. Incidence of uncomplicated clinical malaria and asymptomatic malaria infection were analyzed among these two groups while controlling for confounding factors associated with malaria risk in random effects negative binomial and logistic regression models, respectively.

**Results:**

A total of 3,812 person-trimester observations of 199 adults at least 15 years of age were analyzed. Clinical malaria attacks accounted for 6.3% both in collectors and non-collectors, and asymptomatic malaria infections accounted for 21% and 20% in collectors and non-collectors, respectively. A non-significant lower risk of malaria was observed in the collector group in comparison with the non-collector group after adjusting for other risk factors of malaria and endemicity level (Clinical malaria: adjusted incidence rate ratio = 0.89; 95% confidence interval = 0.65-1.22; p= 0.47).

**Conclusion:**

Being a mosquito collector in Dielmo was not significantly associated with an increased risk of malaria both under holoendemic, mesoendemic and hypoendemic conditions of malaria epidemiology. This result supports the view that HLC, the most accurate method for evaluating malaria transmission, may be used without health concerns in Dielmo.

## Introduction

Identifying the species of *Anopheline* mosquitoes involved in *Plasmodium* transmission and knowledge of their biology are key issues to control malarial vectors [1]. Several entomologic techniques are used to study vector aggressiveness and to evaluate malaria transmission [2, 3]. Human landing catches (HLC) represent the most frequently used method to evaluate human exposure to mosquito bites [4]. HLC allows a direct measurement of the aggressiveness of *Anopheles* mosquitoes on an hourly basis during biting times [1, 4]. Data on the biting frequency of *Anopheles* and the nightly composition of species are helpful in evaluating the efficacy and implementing mosquito control strategies.

However, this method presents some limitations. Indeed, the results obtained using the HLC technique may depend on the skills and the attractiveness of the collector [2, 5–7]. In addition, this technique could pose safety concern as the collectors may be exposed to a potential risk of mosquito-borne disease transmission [8]. To address these limitations, some other techniques such as light traps (CDC miniature light traps) [9] or indoor resting catches by aspiration or spraying have been developed, but these alternative methods are less accurate at estimating malaria transmission than HLC and are subject to potentially large bias [5, 10–15]. Therefore, HLC remains the referent method for evaluating malaria transmission. However, few data are available on the risk of malaria among the collectors. To our knowledge, the only study which evaluates the risk of malaria among mosquito collectors showed that the incidence of the disease was lower in collectors than non-collectors when chemoprophylaxis was provided to collectors [16]. However, collectors of mosquitoes have not used prophylaxis in many studies using HLC [12, 13, 17, 18]. This situation has created debate on the risk of malaria among collectors in the absence of prophylaxis. Because the intensification of malaria prevention methods such as long-lasting insecticide-treated bed nets (LLINs) has decreased human vector contacts [19–22], immunity to *Plasmodium* has declined [19], increasing the risk for adults to develop a clinical attack when bitten by infected mosquitoes. Thus, collector risk could become an issue that need to be evaluated.

The aim of this study was to investigate whether adult mosquito collectors who participated in the Dielmo project in Senegal between 1990 and 2011 have suffered an increased incidence of malaria compared with other villagers.

## Methods

### The study site

Dielmo, the study site, is a Senegalese village with endemic malaria. The village is located 270 km southeast of Dakar (capital of Senegal) in the Sudanian savannah region of central Senegal. It is situated on the marshy bank of a small permanent stream (the Nema) that permits *Anopheline* breeding sites to persist year round. Malaria transmission is intense and occurs throughout the year, with an average of 263 and 120 infected bites per person per year during the periods of 1990–2006 and 2007–2011, respectively [23]. However, the epidemiology of malaria had changed significantly in this village, from holoendemic in 1990 [24] to hypoendemic in 2010 [23].

### Participants and procedure

From July 1990 to December 2011, a longitudinal study was conducted in Dielmo to evaluate malaria morbidity among adult men 15 years of age or greater who were volunteers for collecting mosquitoes compared with those who were non-collectors. Passive and active surveys including the daily monitoring of the inhabitants of Dielmo were used to identify fever episodes and malaria attacks among residents. An individual was considered to be a quarterly resident if he spent at least 75% of his time in Dielmo during that trimester.

If patients developed a fever, they were referred to a health center that was open 24 hours a day, 7 days a week, and were examined by a nurse. Thick smears stained with Giemsa were performed in patients with a fever, and the presence of a parasite on thick smears was determined using light microscopy. Clinical malaria attacks were treated appropriately and the efficacy of the treatment was monitored by daily clinical surveillance of patients and using at least one parasitic control between day 7 and day 35 post-treatment [20]. During the study period, malaria attacks were treated in Dielmo from July 1990 to December 1994 with oral Quinimax, from January 1995 to October 2003 with chloroquine, from November 2003 to May 2006 with amodiaquine (AQ) plus sulfadoxine-pyrimethamine (SP) and from June 2006 until now with ACT (AQ plus artésunate (AS)). However, clinical malaria attacks lasted only a few hours in adults who were permanent residents of Dielmo [25]. The LLINs were offered to all villagers in July 2008 and were renewed in July 2011.

Cross-sectional surveys were also conducted each trimester of the year to assess asymptomatic carriage. Thick smears were performed for all inhabitants enrolled in the Dielmo project and who were present during the cross-sectional survey.

Hemoglobin and ABO blood typing measurements were systematically conducted for each individual enrolled in the Dielmo project at study inclusion and were maintained in a biological databank; information about sex, age and being born in Dielmo was also maintained at the facility.

### Study population

In our study, we focused on person-trimester observations over 4 different periods, each of these periods being associated with major changes in malaria epidemiology [23]. The first period of observation was from July 1990 to December 1994 and corresponds to the oral Quinimax treatment period. The second period was between April 1999 and June 2003, and chloroquine was used during this period. From April 2005 to June 2008, corresponding to the third period of observation, the amodiaquine (AQ)/sulfadoxine-pyrimethamine (SP) combination was used, followed by ACT (AQ+AS). The last period was from July 2008 to December 2011 when ACT treatment was associated with LLINs. All adult men aged 15 years and older who were enrolled in the Dielmo project and who were quarterly residents (having spent at least 75% of the trimester’s days in Dielmo) were included in this study.

Written informed consent was obtained from all participants. The study was approved by the Ministry of Health of Senegal and the assembly of the Dielmo population.

### Procedure of mosquito collection by human landing catches

The HLC method consists of sitting down with legs exposed and waiting for mosquitoes to come feed on collectors. Every month in Dielmo, HLC was performed inside the concession (indoor HLC) and outside the concession (outdoor HLC). For each indoor HLC, two collectors were involved in mosquitoes catching. The same method was used for outdoor HLC. The households selected for the catches remained unchanged during the study. The mosquito collections were performed over three consecutive nights during the first week of every month (average of 12 person-nights of capture per night). The HLC was performed between 7 PM and 7 AM. The period of collection was divided into 1-hour segments; thus, the two volunteers worked for one hour and rested for one hour alternately [5]. The Dielmo health center provided medical supervision for all the collectors as for the other members of the community.

### Outcome and independent variables definition

The analysis was based on person-trimester observations. For each trimester, the incidence of clinical malaria and non-malaria fever, and the prevalence of *Plasmodium* asymptomatic infections were compared between collectors and non-collectors. Clinical malaria cases were defined as individuals who had signs or symptoms of fever associated with a parasite density above an age and endemicity dependent threshold [26]. Non-malaria fever cases were defined as individuals who had signs or symptoms of fever associated with a parasite density below the threshold. The outcome variable was the number of clinical malaria attacks and the number of episodes of non-malaria fever per trimester, and the results (positive or negative) of thick blood smears in asymptomatic individuals, among collectors and non-collectors. The incidence rates of the clinical malaria attacks were calculated as the ratio of the number of clinical malaria attacks recorded divided by the number of person-days of follow-up during a given period. We derived the mean monthly and yearly incidence rates by period from the daily incidence rates based on 30.4 and 365.25 days per month and per year, respectively.

The following variables were investigated in the analysis: being mosquito collectors, the number of collection nights, type of hemoglobin, ABO blood group, age group (defined in 3 groups as follows: 15–29 years old, 30–44 years old and 45 years and older), period of treatment, being born in Dielmo, the trimester of the year and the entomological inoculation rate (EIR). Each variable was analyzed separately using simple regression analysis to assess the association with malaria risk.

Random-effect negative binomial regression models and random-effect logistic regression models, were used to analyze the number of clinical episodes (*i*.*e*. clinical malaria or non-malaria fever), and the prevalence of asymptomatic malaria infection, respectively, taking into account the interdependence of successive observations in the same individuals.

Variables that were P<0.2 in simple regression analyses were integrated in multiple regression analyses. Step-wise elimination of variables was performed based on the AIC (Akaike information criterion) in the model. The significance level was fixed at α = 0.05 in the final model.

Analyses were performed using Stata Software version 11.0 (College Station, Texas, USA) and R software software version 3.1.2 [27] and the packages MASS, lme4 and Epi.

## Results

### Observations among mosquito collectors and non-collectors

A total of 3,812 person-trimester observations (339,313 person-days) were analyzed with 1,372 (36%; 121,859 person-days) observations from mosquito collectors and 2,440 (64%; 217,454 person-days) observations from non-collectors. A total of 240 malaria cases were observed during the study period. Malaria attacks accounted for 6.3% both in collectors (n = 86) and non-collectors (n = 154). Twenty one percent (n = 286) and 20% (n = 481) of asymptomatic thick smears were positive in collectors and non-collectors, respectively. Episodes of non-malaria fever accounted for 25.1% in collectors (n = 345) and 19.7% in non-collectors (n = 481). The number of nights of being a mosquito collector varied from 1 time to 7 times per trimester per mosquito collector. Table 1 shows the socio-demographic, biological and other characteristics of the studied population.

**Table 1:** Socio-demographic and others characteristics among mosquito collectors and non-collectors in Dielmo (n = 3,812) using a random-effect logistic regression.

Characteristics	Non-collectors (%)	Collectors (%)	OR (95% CI)	*P*-value
**No. person-trimester under survey (100%)**	2,440	1,372		
**Malaria attacks**				
No	2,286 (93.7)	1,286 (93.7)	1	
Yes (at least one)	154 (6.3)	86 (6.3)	1.09 (0.75–1.58)	0.66
**Number of malaria attacks**				
0	2,286(93.7)	1,286 (93.7)	1	
1	134 (5.5)	84 (6.1)	1.16 (0.79–1.70)	0.45
2 and more	20 (0.8)	2 (0.2)	0.29 (0.04–1.90)	0.20
**Asymptomatic malaria infections**				
Without	1,959 (80)	1,086 (79)	1	
With	481 (20)	286 (21)	1.004 (0.81–1.25)	0.97
**Number of episodes of non-malaria fever**				
0	1,959 (80)	1,027 (75)	1	
1	401 (16)	268 (20)	1.25 (0.99–1.56)	0.06
2	62 (3)	61 (4)	2.1 (1.32–3.35)	<0.01
3 or more	18 (1)	16 (1)	1.65 (0.71–3.81)	0.24
**Socio-demographic characteristics**				
**Age group**				
15–29 years old	953 (30)	662 (48)	1	
30–44 years old	630 (26)	372 (27)	0.38 (0.27–0.52)	<0.01
45 years old and over	857 (35)	338 (25)	0.14 (0.09–0.21)	<0.01
**Born at Dielmo**				
No	566 (23)	171 (13)	1	
Yes	1,815 (74)	1,201 (88)	3.94 (1.89–8.23)	<0.01
**Biological characteristics**				
**HB type**				
AA	2,136 (88)	1,194 (87)	1	
AS	240 (10)	161 (12)	0.93 (0.33–2.68)	0.90
AC	39 (2)	15 (1)	0.77 (0.04–15.68)	0.87
**ABO Group**				
O	817 (33)	456 (33)	1	
A	835 (34)	481 (35)	0.98 (0.43–2.23)	0.95
B	644 (26)	384 (28)	1.53 (0.65–3.61)	0.33
AB	93 (4)	38 (3)	1.39 (0.28–6.97)	0.69
**Others characteristics**				
**Treatment period**				
Quinimax	736 (30)	368 (27)	1	
Chloroquine	543 (22)	463 (34)	2.84 (2.17–3.73)	<0.01
AQ+SP/ACT	522 (21)	198 (14)	0.41 (0.30–0.55)	<0.01
ACT+LLINs	639 (26)	343 (25)	0.66 (0.49–0.87)	<0.01
**Quarter of the year**				
First quarter of the year	566 (23)	275 (20)	1	
Second quarter of the year	619 (25)	329 (24)	1.2 (0.94–1.54)	0.15
Third quarter of the year	652 (27)	388 (28)	1.34 (1.04–1.71)	0.02
Fourth quarter of the year	603 (25)	380 (28)	1.39 (1.08–1.77)	0.01

OR: Odds ratio.

Some variables differed between the two groups (Table 1), while the incidence of malaria attacks, malaria asymptomatic infection and biological variables (ABO blood group and hemoglobin type) did not differ significantly between the two groups.

Compared with non-collectors (74%), most of the mosquito collectors (88%) were born in Dielmo (odds ratio [OR] = 3.94; 95%CI = 1.89–8.23; p<0.01), and collectors were younger than non-collectors (p<0.01). The number of episodes of non-malaria fever was significantly higher among collectors than in non-collectors in simple regression analysis (p<0.01).

### Incidence density of clinical malaria attacks over the study period and according to age group

The overall incidence rates of malaria over the study period were 0.26 and 0.30 attacks per person per year among mosquito collectors and non-collectors, respectively.

**Fig 1:**
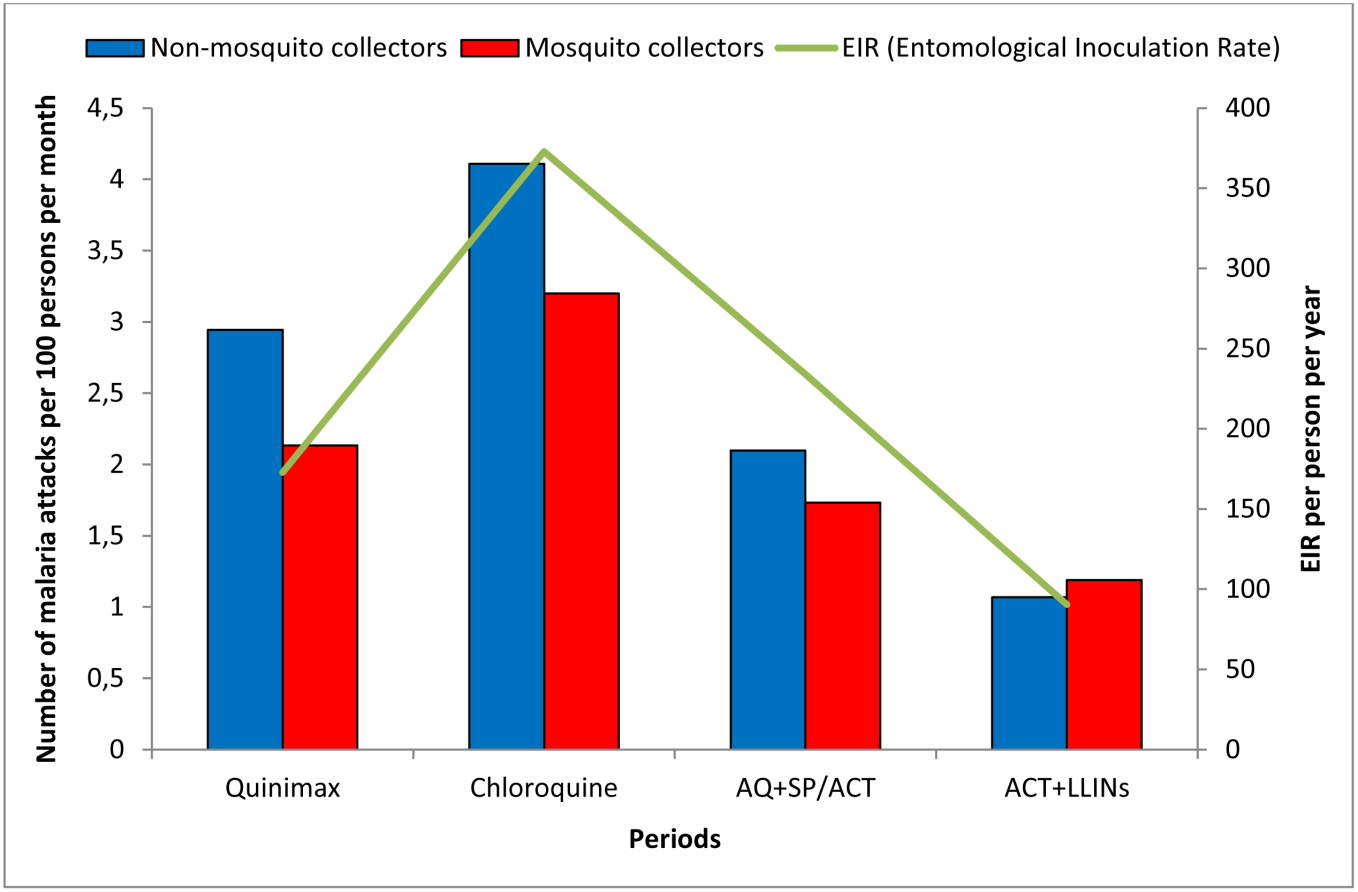
Malaria incidence among mosquito collectors and non-mosquito collectors according to different periods in Dielmo.

The malaria incidence rates varied across treatment periods but did not significantly differ between the two groups during each treatment period (Fig 1). Indeed, the incidence density was slightly higher among non-collectors than mosquito collectors before the LLINs were implemented back when the prevalence of malaria was high (an average of 35% in adults of the study population). However, the incidence density was similar between the two groups during the period of the association of ACT and LLINs, and this observation coincided with both the decrease in malaria prevalence (an average of 4%) and the entomological inoculation rate (EIR). During the oral Quinimax treatment period, the prevalence of malaria was an average of 47%, and the malaria incidence rates were 2.95 and 2.13 attacks per 100 persons per month in non-collectors and mosquito collectors, respectively. During the period corresponding to the implementation of ACT+LLINs, malaria incidence decreased in these two populations to 1.07 and 1.19 attacks per 100 persons per month in non-collectors and collectors, respectively.

**Fig 2:**
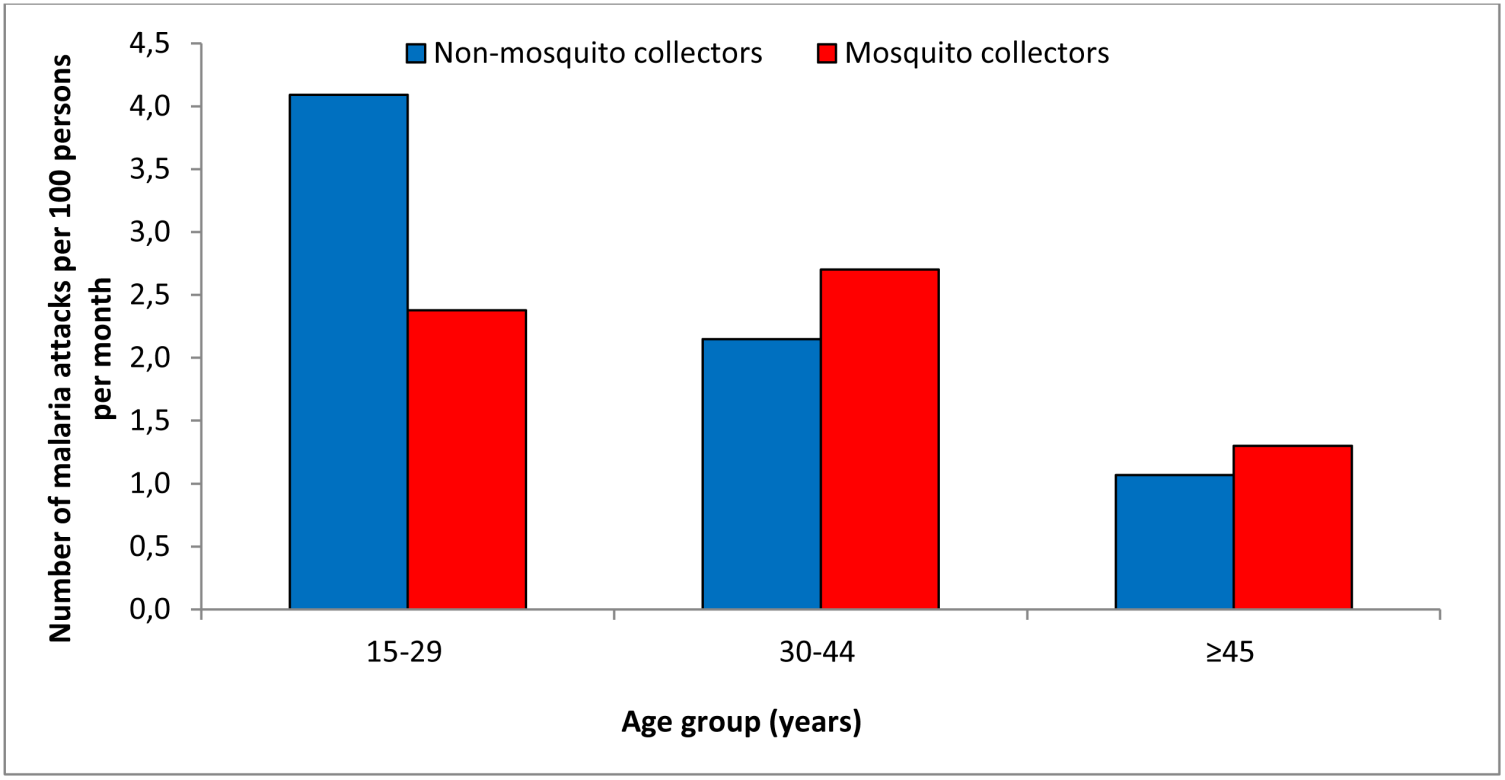
Malaria incidence among mosquito collectors and non-mosquito collectors according to age group in Dielmo.

According to age group, the malaria incidence rate was similar among collectors and non-collectors, except for the age group of 15–29 years (Fig 2). Indeed, the malaria incidence density was 1.7 times lower in collectors compared with non-collectors (2.4 versus 4.1 attacks per 100 persons per month) in adults aged 15–29 years old, and this difference was significant (χ^2^ = 9.9; p<0.01).

### Factors associated with clinical malaria attacks among collectors and non-collectors

Table 2 describes both individual characteristics according to the incidence of at least one clinical malaria attack and the results of the simple and multiple regression analyses. In simple regression analyses, being a collector was not significantly associated with clinical malaria risk (Incidence rate ratio [IRR] = 0.97; 95%CI = 0.71–1.33; p = 0.85). Similarly, the number of the collection nights during the quarter was not associated with clinical malaria risk. The type of hemoglobin and blood group variables were not associated significantly with increased clinical malaria risk. However, being born in Dielmo (IRR = 0.32; 95%CI = 0.19–0.54; p<0.01), combination therapy period and the deployment of LLINs were associated with a significant decrease in the number of clinical malaria attacks. Older adults had fewer clinical malaria attacks compared with younger adults (IRR = 0.59; 95%CI = 0.41–0.87; p<0.01 and IRR = 0.27; 95%CI = 0.16–0.45, p<0.01, respectively, for adults aged 30–44 years and 45 years and older).

**Table 2:** Socio-demographic and biological characteristics according to malaria attacks and results of random-effect negative binomial regression models exploring factors associated with clinical malaria cases (n = 3,812).

Characteristics	Subcategory	Number of persons n = 199 (100%)	Number of person-trimester n = 3,812 (100%)	Clinical malaria cases		Simple regression analysis		Multiple regression analysis	
				No n = 3,572 n (%)	Yes n = 240 n (%)	IRR (95% CI)	*P*-value	aIRR (95% CI)	*P*-value
**Mosquitoes collectors**	No	-	2,440 (64)	2,286 (64)	154 (64)	1		1	
	yes	-	1,372 (36)	1,286 (36)	86 (36)	0.97 (0.71–1.33)	0.85	0.89 (0.65–1.22)	0.47
**Number of nights of collections**	0	-	2,440 (64)	2,286 (64)	154 (64)	1			
	1	-	461 (12)	430 (12)	31 (13)	0.94 (0.62–1.42)	0.77		
	2	-	445 (12)	418 (12)	27 (11)	0.94 (0.60–1.48)	0.80		
	3 and more	-	466 (12)	438 (12)	28 (12)	1.05 (0.67–1.66)	0.83		
**Age group**	15–29 years old	-	1,615 (42)	1,478 (41)	137 (57)	1		1	
	30–44 years old	-	1,002 (26)	937 (27)	65 (27)	0.59 (0.41–0.87)	<0.01	0.59 (0.40–0.88)	<0.01
	45 years old and over	-	1,195 (31)	1,157 (32)	38 (16)	0.27 (0.16–0.45)	<0.01	0.31 (0.18–0.53)	<0.01
**Born at Dielmo**	No	65 (33)	796 (21)	714 (20)	82 (34)	1		1	
	Yes	134 (67)	3,016 (79)	2,858 (80)	158 (66)	0.32 (0.19–0.54)	<0.01	0.38 (0.24–0.61)	<0.01
**Biological characteristics**									
**HB type**	AA	167 (84)	3,330 (87)	3,120 (87)	210 (88)	1			
	AS	22 (11)	401 (11)	381 (11)	20 (8)	0.82 (0.39–1.76)	0.62		
	AC	2 (1)	54 (1)	51 (1)	3 (1)	0.50 (0.06–4.15)	0.52		
**ABO Group**	O	63 (32)	1,273 (33)	1,187 (33)	86 (36)	1			
	A	57 (29)	1,316 (35)	1,224 (34)	92 (38)	1.08 (0.61–1.91)	0.80		
	B	48 (24)	1,028 (27)	978 (27)	50 (21)	0.70 (0.37–1.32)	0.27		
	AB	9 (5)	131 (3)	126 (4)	5 (2)	0.59 (0.15–2.29)	0.45		
**Others characteristics**									
**Treatment period**	Quinimax	104	1,104 (29)	1,031 (29)	73 (30)	1		1	
	Chloroquine	106	1,006 (26)	913 (26)	93 (39)	1.07 (0.76–1.49)	0.70	1.16 (0.77–1.75)	0.49
	AQ+SP/ACT	110	720 (19)	678 (19)	42 (18)	0.58 (0.38–0.88)	0.01	0.74 (0.48–1.16)	0.19
	ACT+LLINs	119	982 (26)	950 (27)	32 (13)	0.28 (0.18–0.45)	<0.01	0.43 (0.27–0.70)	<0.01
**Quarter of the year**	First quarter of the year	166	841 (22)	801 (22)	40 (17)	1		1	
	Second quarter of the year	169	948 (25)	899 (25)	49 (20)	0.98 (0.66–1.46)	0.92	0.96 (0.65–1.42)	0.84
	Third quarter of the year	182	1,040 (27)	963 (27)	77 (32)	1.41 (0.97–2.04)	0.07	1.31 (0.88–1.95)	0.19
	Fourth quarter of the year	173	983 (25)	909 (25)	74 (31)	1.53 (1.06–2.20)	0.02	1.47 (1.02–2.13)	0.04
**EIR (Entomological Inoculation Rate per person-trimester)**		-				1.007 (1.004–1.01)	<0.01	1.003 (0.999–1.007)	0.10

IRR: Incidence rate ratio; aIRR: adjusted incidence rate ratio.

Most of the simple regression analysis results were confirmed in the final multiple regression model. After adjusting for potential confounders, being a collector was not significantly associated with a lower risk of clinical malaria attacks (adjusted incidence rate ratio [aIRR] = 0.89; 95%CI = 0.65–1.22; p = 0.47). Control variables of being born in Dielmo (aIRR = 0.38; 95%CI = 0.24–0.61, p<0.01) and the ACT+LLINs implementation period (aIRR = 0.43; 95%CI = 0.27–0.70, p<0.01) were significantly associated with a lower risk of clinical malaria attacks. Similarly, older adults had fewer clinical malaria attacks than younger adults (aIRR = 0.59; 95%CI = 0.40–0.88; p<0.01 and aIRR = 0.31; 95%CI = 0.18–0.53; p<0.01, respectively, for adults aged 30–44 years and 45 years and older).

### Factors associated with asymptomatic malaria infection among collectors and non-collectors

Table 3 shows the results of simple and multiple regression analyses according to asymptomatic malaria infection. In simple regression analyses, being a collector was not significantly associated with asymptomatic malaria infection (OR = 1.03; 95%CI = 0.85–1.26; p = 0.73). Similarly, the number of the collection nights during the quarter and being born in Dielmo were not associated with the presence of asymptomatic malaria infection. The type of hemoglobin and blood group variables were not associated significantly with asymptomatic malaria infection. However, the treatment based on chloroquine period, combination therapy period and the deployment of LLINs were associated with a significant decrease of asymptomatic malaria infection. Older adults were less infected compared with younger adults (OR = 0.33; 95%CI = 0.25–0.43; p<0.01 and OR = 0.21; 95%CI = 0.15–0.30, p<0.01, respectively, for adults aged 30–44 years and 45 years and older). The EIR, the third and fourth trimesters of the year were associated with an increased risk of asymptomatic malaria infection. In the final multiple regression analyses, being a collector was not significantly associated with a lower risk of asymptomatic malaria infection (aOR = 0.88; 95%CI = 0.71–1.09; p = 0.23). Older adults remained less infected compared with younger adults (aOR = 0.40; 95%CI = 0.30–0.52; p<0.01 and aOR = 0.38; 95%CI = 0.28–0.52, p<0.01, respectively, for adults aged 30–44 years and 45 years and older). The combination therapy period and the deployment of LLINs were associated with a significant decrease of asymptomatic infection whereas the EIR and the third trimester of the year were associated with an increase of asymptomatic malaria infection. The type of hemoglobin AC was associated with an increased risk of asymptomatic malaria infection.

**Table 3:** Random-effect logistic regression models exploring factors associated with asymptomatic malaria infection (n = 3,812).

Characteristics	Subcategory	Simple regression analysis		Multiple regression analysis	
		OR (95% CI)	*P*-value	aOR (95% CI)	*P*-value
**Socio-demographic characteristics**					
**Mosquitoes collectors**	No	1		1	
	yes	1.03 (0.85–1.26)	0.73	0.88 (0.71–1.09)	0.23
**Number of nights of collections**	0	1			
	1	0.90 (0.68–1.19)	0.47		
	2	1.08 (0.82–1.43)	0.59		
	3 and more	1.16 (0.88–1.53)	0.29		
**Age group**	15–29 years old	1		1	
	30–44 years old	0.33 (0.25–0.43)	<0.01	0.40 (0.30–0.52)	<0.01
	45 years old and over	0.21 (0.15–0.30)	<0.01	0.38 (0.28–0.52)	<0.01
**Born at Dielmo**	No	1			
	Yes	1.17 (0.83–1.65)	0.37		
**Biological characteristics**					
**HB type**	AA	1		1	
	AS	1.04 (0.66–1.64)	0.85	0.73 (0.48–1.11)	0.15
	AC	2.45 (0.75–8.03)	0.14	3.15 (1.08–9.19)	0.04
**ABO Group**	O	1			
	A	0.81 (0.57–1.17)	0.26		
	B	1.02 (0.70–1.49)	0.92		
	AB	0.69 (0.31–1.51)	0.35		
**Treatment period**	Quinimax	1		1	
	Chloroquine	0.61 (0.49–0.76)	<0.01	0.65 (0.49–0.84)	<0.01
	AQ+SP/ACT	0.20 (0.14–0.27)	<0.01	0.26 (0.19–0.35)	<0.01
	ACT+LLINs	0.03 (0.02–0.05)	<0.01	0.05 (0.03–0.07)	<0.01
**Quarter of the year**	First quarter of the year	1		1	
	Second quarter of the year	1.26 (0.98–1.62)	0.07	1.29 (0.99–1.68)	0.06
	Third quarter of the year	1.57 (1.23–2.00)	<0.01	1.48 (1.11–1.97)	<0.01
	Fourth quarter of the year	1.32 (1.03–1.69)	0.03	1.29 (0.99–1.69)	0.06
**EIR (Entomological Inoculation Rate per person-trimester)**		1.008 (1.006–1.009)	<0.01	1.003 (1.00–1.01)	0.023

OR: Odds ratio; aOR: adjusted odds ratio.

### Incidence of episodes of non-malaria fever among collectors and non-collectors

The overall incidence rates of episodes of non-malaria fever over the study period were 1.32 and 0.98 non-malaria fever episodes per person per year among mosquito collectors and non-collectors, respectively. Controlling for the effects of age, trimester and period of treatment, the incidence of episodes of non-malaria fever was not significantly higher among collectors than non-collectors (aIRR = 1.07; 95%CI: 0.92–1.25; p = 0.37).

## Discussion

This study was conducted to evaluate whether collecting mosquitoes using the HLC technique increased the risk of malaria attacks in Dielmo villagers. HLC is considered to be the gold standard method for monitoring human exposure to *Anopheles* and malaria transmission, but it could pose a safety concern. Although the risk of malaria among mosquito collectors has been debated over decades [8], no study to date had investigated this important issue.

Our study showed that there was no significant difference in Dielmo in the risk of malaria in mosquito collectors compared with non-collectors. This finding was consistently observed over a 20-year period that was marked by drastic changes in malaria endemicity, which switched from holoendemicity at the beginning of the project to mesoendemicity when the drug policy switched from chloroquine to combination therapy and to hypoendemicity after the deployment of LLINs [23]. There was also no significant difference in the incidence of episodes of non-malaria fever between the two groups, suggesting that collectors were not at higher risk of other mosquito-borne diseases that cause fever, *e*.*g*. arbovirosis.

Independent of the collector / non-collector status of each villager and the endemicity period, the following two factors affected the risk of malaria attacks among adults: age group, whereby the highest incidence was observed in younger adults, and duration of residence in Dielmo. The lowest incidence of malaria was observed in people exposed since birth to the perennial transmission of malaria observed in Dielmo. Mosquito collectors were younger than non-collectors, thus increasing malaria risk, but collectors were more frequently born in Dielmo, thus decreasing malaria risk. Both potential confounding factors were controlled in the analysis, and there was no significant difference in malaria risk between collectors and non-collectors.

In fact, before the deployment of LLINs, it was not surprising that collecting mosquitoes did not increase the risk of malaria. When working, collectors catch most mosquitoes when they land and thus before they bite. Concomitantly, other adults in the village are probably more exposed to malaria than mosquito collectors since they don’t use mosquito nets that night. For mosquito collectors, in most cases, the risk of receiving an infected bite was lower the night they collected mosquitoes compared with other nights.

Interestingly, during the most recent period where most villagers were using LLINs each night and where malaria risk dramatically decreased in Dielmo villagers [23], the risk of malaria attacks remained similar in mosquito collectors compared with non-collectors. During this period, acquired immunity in adults decreased markedly in all age groups [19, 20]; thus, both collectors and non-collectors were much more at risk of an acute clinical attack from each infected mosquito bite, which was independent of age and duration of residence in Dielmo. The fact that the clinical malaria incidence was similar in collectors and non-collectors after the deployment of LLINs suggests that either the skills of the collectors allowed them to consistently collect mosquitoes when landing before they bite, thus preventing malaria risk, or that the balance of risk was negligible when accounting for changes in mosquito feeding behavior after the deployment of LLINs. In particular, *Anopheles gambiae* s.l. and *A*. *funestus* became more exophilic and bit much earlier in the night [28], or even bit during the day [29]. These changes in vector behavior may have concentrated most remaining malaria transmission before adults began to sleep under nets or to collect mosquitoes, and thus tend to homogenize risks for collectors and non-collector adults. These two hypotheses are not exclusive of each other and both may have contributed to the lack of difference in incidence of malaria between collectors and non-collectors.

## Conclusion

Being a mosquito collector in Dielmo was not significantly associated with an increased risk of malaria (or non-malaria fever) under holoendemic, mesoendemic or hypoendemic conditions of malaria epidemiology, i.e. when inhabitants were highly, moderately or weakly naturally protected against malaria. Whatever the usual level of protection of collectors and non-collectors, being a mosquito collector could not be considered as an activity at risk of malaria or non-malaria fever in Dielmo. This result supports the view that HLC, the most accurate method for evaluating malaria transmission, may be used without health concerns in malaria endemic areas when the collectors are local residents. However, further data are needed to confirm these data in other settings.
